# Beneficial effects of relaxin on motility characteristics of stored boar spermatozoa

**DOI:** 10.1186/s12958-015-0021-4

**Published:** 2015-03-31

**Authors:** Jean M Feugang, Juan C Rodríguez-Muñoz, Darby S Dillard, Mark A Crenshaw, Scott T Willard, Peter L Ryan

**Affiliations:** Facility for Organismal and Cellular Imaging (FOCI), Department of Animal and Dairy Sciences, Mississippi State University, Mississippi State, Mississippi, 39762 USA; Department of Biochemistry and Molecular Biology & Entomology and Plant Pathology, Mississippi State University, Mississippi State, Mississippi, 39762 USA; Department of Pathobiology and Population Medicine, Mississippi State University, Mississippi State, Mississippi, 39762 USA

**Keywords:** RXFP1, RXFP2, Immunofluorescence, Relaxin receptor, cAMP, Motility, Sperm storage, Semen

## Abstract

**Background:**

Relaxin is detected in seminal plasma of many species and its association with sperm motility may be beneficial in some aspects of assisted reproduction. Here, we immunolocalized relaxin receptors and investigated the effects of exogenous relaxin on motility characteristics, viability, and cAMP content of boar spermatozoa after storage.

**Methods:**

Commercial doses of boar semen were obtained on the collection day (Day 0) and kept in shipping containers at room temperature for up to 4 days (Day 4). On Day 0, spermatozoa were fixed for immunofluorescence detection of relaxin receptors RXFP1 and RXFP2 (Experiment 1). Semen aliquots were taken from the same dose at Day 0, Day 1, and Day 2 (Experiment 2a), and Day 2 and Day 4 (Experiment 2b) for analyses. Alive spermatozoa were purified and incubated (1 h-37°C) with 0, 50, or 100 ng relaxin/ml (Experiment 2a) and 0, 100, or 500 ng relaxin/ml (Experiment 2b). Afterward, aliquots of each treatment group were subjected to motility (Experiments 2), viability (Experiment 3) analyses, and cAMP quantification (Experiment 4). Data (3–4 independent replicates) were statistically analyzed (ANOVA followed by pairwise comparisons) and p values less or equal to 0.05 was set for significant difference.

**Results:**

Both RXFP1 and RXFP2 receptors were immunolocalized on the entire spermatozoon. Relaxin concentration of 100 ng/ml significantly improved the proportions of motile, progressive, and rapid spermatozoa up to Day 2. Only 500 ng relaxin/ml provided beneficial effects on Day 4. The viability of spermatozoa was not affected by relaxin (100 ng/ml) during storage, but the extent of mitochondria membrane damages was significantly decreased. Furthermore, relaxin did not affect the cAMP contents of spermatozoa during storage, in our conditions.

**Conclusions:**

Relaxin could be a valuable motility booster of stored- or aged-spermatozoa for assisted reproduction techniques. However, the related-intracellular signaling cascades of relaxin in boar spermatozoa remain undetermined.

**Electronic supplementary material:**

The online version of this article (doi:10.1186/s12958-015-0021-4) contains supplementary material, which is available to authorized users.

## Background

Relaxin is a polypeptide of about 6 kDa discovered decades ago and also known as a hormone of pregnancy in many mammals [1,2]. It has been described as a member of the insulin family proteins found in various reproductive and non-reproductive tissues and having pleiotropic functions in both males and females [2-4]. The effects of relaxin in females are well-documented, especially during early (e.g., implantation and placentation) and late (e.g., growth and softening of the uterine cervix) pregnancy [3], but its roles in male reproductive organs are still not fully understood [2,5,6].

Various reproductive tissues of males express relaxin and, depending on the species, the prostate and/or the testes appear as the main sources of its production [5,6]. The prostate is well-established as the major site of relaxin production in humans, primates, and rats [6,7], whereas testes appear as the major source in dogfish sharks [8] and boars [9,10]. Despite these differences and regardless of the species, relaxin secretions are found in seminal plasma and its levels are associated with sperm motility of semen ejaculates [11-14]. Indeed, positive correlations have been found between immunoreactivity levels of seminal relaxin and sperm motility in both humans and animals [11,14-19], which findings are consistent with other studies reporting decreased sperm motility in seminal plasma preparations supplemented with relaxin antiserum [20,21]. Additionally, experimental studies conducted with men and boars spermatozoa demonstrated direct stimulatory effects of relaxin on sperm motility [21-24], while revealing further effects of relaxin on mitochondrial function, capacitation, acrosome reaction, cAMP production, and calcium accumulation in spermatozoa [23,25].

It is likely that the aforementioned effects of relaxin are results of its interaction with specific plasma membrane receptors that have been characterized in various reproductive and non-reproductive tissues [6]. Indeed, relaxin has been described as the endogenous ligand of the membrane G-protein coupled receptor, RXFP1 (LGR7); however, it is still able to bind, at lower affinity, to the closely associated receptor, RXFP2 (LGR8). Both receptors have been differentially detected in mature spermatozoa of boars [26] and men [25], as well as in reproductive tracts of rats [27], boars [10], and monkeys [28] through, almost exclusively, indirect technical approaches. The interaction of relaxin with its receptor RXFP1 has the potential to activate a variety of intracellular pathways in a possible cell type-dependent manner, which leads to the production of cAMP generally known as the major intra-cellular transducer of relaxin in somatic cells [29]. At present, the knowledge of the physiological effects of relaxin on spermatozoa of many species is still limited [25].

From this background, it appears that the presence of relaxin in seminal plasma or semen preparation is favorable to sperm function and progression within the female genital tract [17]. Therefore, the ability of relaxin to sustain or restore sperm motility may be beneficial in assisted reproduction procedures; especially in situations of reduced sperm motility or unanticipated delayed insemination, using either fresh or frozen-thawed semen. Thus, the supplementation of semen preparation with relaxin can be a conceivable way to reduce the pace of the progressive decline of semen quality during or after storage (e.g., extended transportation). Therefore, we hypothesized that relaxin supplementation to stored- or aged-semen prior to insemination may boost sperm motility with the expectation to uphold satisfactory fertility rates. Here, we tested this hypothesis with commercial doses of proven fertile boar semen by assessing the presence of RXFP1 and RXFP2 membrane receptors on freshly collected spermatozoa through direct immunofluorescence (*i*), followed by the evaluation of motility characteristics (*ii*), viability (*iii*), and quantification of the cyclic AMP (cAMP) content (*iv*) of spermatozoa stored for up to 4 days, then exposed to porcine relaxin (pRLX).

## Methods

### Sperm preparation

Semen was collected from fertile boars at a commercial farm (Prestage Farms Inc., West Point, MS) and diluted for a short-term preservation of up to 3 days in the Beltsville Thawing Solution (BTS; Minitube of America, Mount Horeb, WI). Insemination doses were prepared at the boar stud by pooling semen of 2 to 4 individual boars diluted with BTS. Purchased doses were wrapped with ice cold packs and immediately shipped to our laboratory, within 2–3 hours post collection. Semen doses (80 ml each) were stored in our laboratory for up to 5 days, at approximately 16–19°C in the Styrofoam shipping container. Four independent doses of semen (n = 4) that were prepared from different boars were used for each experimental replicate. A given boar semen was not used in more than one dose. Semen aliquots were taken from the same dose at Day 0, Day 1, Day 2, or Day 4 post-semen collection, corresponding to 0, 1, 2, or 4 days of storage, respectively.

Aliquots were centrifuged (250 g for 5 min) at room temperature to remove the extender. Resulted sperm pellets were dispersed in approximately 500 μl of pre-warmed 0.01 M phosphate buffer saline (PBS, pH7.4: 0.138 M NaCl, 0.0027 M KCl; Sigma-Aldrich, Saint Louis, MO) supplemented with 1 mg PVP-40 per milliliter of PBS (PBS/PVP). Sperm suspensions were placed onto a one-layer Percoll gradient (PorciPure, Nidacon; Mölndal, Sweden) for a centrifugation (600 g - 30 minutes) that separates motile spermatozoa from debris and other cells (i.e., dead/abnormal sperm, somatic cells, and virus and bacteria if any). Resulting pellets of living spermatozoa were subsequently resuspended in PBS/PVP and centrifuged (250 g - 5 minutes) to remove the remaining Percoll. Sperm pellets were resuspended in a pre-warmed PBS/PVP and their concentrations were evaluated using the SpermaCue Photometer (Minitube of America). All samples were adjusted to 75 × 10^6^ spermatozoa per milliliter and aliquots of one milliliter were used for relaxin treatments, as described below.

### Experiment 1: immunofluorescence detection of RXFP1 and RXFP2 receptors

Polyclonal antibodies raised against the human RXFP1 (H-160; sc-50328; 200 μg/ml) and RXFP2 (H-150; sc-50327; 200 μg/ml) were purchased from Santa Cruz Biotech (Santa Cruz, CA, USA). Both antibodies were selected because of the high identity and similarity (99%) of their immunogenic sequences (amino acids 61–220 for RXFP1 and amino acids 81–230 for RXFP2) with other species (e.g., chimpanzee, cow, dog, horse, human, and mouse; See Additional file 1: Figure S1 and Additional file 2: Figure S2). In a previous study we demonstrated their immunoreactivities with and specificities to porcine tissues [26]. For immunofluorescence, aliquots of Day 0 purified spermatozoa were mixed with 4% methanol-free formaldehyde solution and incubated for 1 hour. Afterward, spermatozoa were spread on histological slides and air-dried. Slides were incubated with the washing buffer containing 0.1% Triton-X100 (30 min), followed by 1 hour incubation with 4% (v/v) normal goat blocking serum. Slides were incubated with 100× diluted RXFP1 and RXFP2 primary antibodies. After an overnight incubation at 4°C, slides were washed and incubated 1 hour with 200x diluted FITC-conjugated secondary antibody (goat anti-rabbit IgG-FITC; sc-2012; 400 μg/ml) and then washed and mounted with a medium containing DAPI. Slides were observed under a Zeiss Confocal Laser Scanning Microscope (LSM 510; Carl Zeiss Micro Imaging GmbH, Jena, Germany). A (DAPI/Fluorescein/Transmission) filter set was used in single channel mode imaging. Excitation wavelengths of 405 nm/488 nm and Band Pass Emission wavelengths of 420–480 nm (Blue) and Long Pass wavelengths of 505 nm (Green) were acquired at 1024 × 1024 pixel formats for imaging purposes. Sperm without primary or secondary antibodies were used as negative controls. All procedures took place at room temperature, unless otherwise indicated. Samples were washed three times with PBS/PVP + 0.1% Tween-20 between steps.

### Experiment 2a: effect of porcine relaxin (pRLX) on sperm motility after short storage

Semen aliquots were prepared as indicated above (75 × 10^6^ spermatozoa/ml) at Day 0, Day 1, Day 2, and Day 4 post-semen collection. Each aliquot was supplemented with 0, 50, or 100 ng/ml of pRLX (porcine corpus luteum extract that was purified, lyophilized, and rehydrated in high pure water for use in this study). Sperm aliquots were subsequently incubated at 37°C for 1 hour. The incubation time was chosen based upon previous studies and the reported loss of relaxin activity overtime [30]. After incubation, all sperm aliquots (75 × 10^6^ spermatozoa/ml) were diluted approximately 5 times and loaded (2 μl) into pre-warmed caffeine-free microscope chamber slides (Standard Count 4-chamber Slide Leja®, 20 micron, Nieuw Vennep, The Netherlands). Three fields per chamber-slide were considered to analyze the motility characteristics of spermatozoa from each treatment group, using a Computer-Assisted Sperm Analyzer or CASA (HTM-IVOS Hamilton-Thorne Biosciences, Version 12.3, Beverly, MA). A total of 48 fields (4 replicates × 4 doses of semen × 3 fields/chamber), averaging (+/− SD) 198 +/− 22 spermatozoa per field were considered for analyses, using pre-set values of the CASA machine (e.g., 60 frames/sec; VAP and STR of progressive cells: 45 μm/sec and 45%, respectively; VAP and VSL cutoffs of slow cells: 20 and 5 μm/sec, respectively; magnification: 1.89×, and temperature of 37°C).

Motility characteristics corresponded to total motile, forward progressive, and rapid and forward progressive (less or equal to 45 μm/sec) spermatozoa, while velocity corresponded to Velocity Straight Line (VSL), Velocity Curve Line (VCL), and Velocity Average Path (VAP). Parameters such as amplitude of lateral head displacement (ALH, in μm), beat cross frequency (BCF, in Hz), straightness (STR; VSL/VAP × 100) and linearity (LIN; VSL/VCL × 100) ratios were also recorded.

### Experiment 2b: effect of porcine relaxin (pRLX) on sperm motility after longer storage

Experiment 2a showed that relaxin concentrations of 100 ng/ml or less have no effects on Day 4. Therefore, experiment 2b was undertaken to test the effects of a higher concentration of relaxin. Sperm aliquots were prepared on Day 4 as indicated above and incubated (for 1 h at 37°C) with final concentrations of 0, 100, or 500 ng relaxin/ml. Then after, spermatozoa were subjected to motility analyses as indicated in experiment 2a. The above mentioned motility characteristics were evaluated on approximately 7,200 +/− 256 spermatozoa in 36 fields (3 replicates × 4 doses of semen × 3 fields/chamber) analyzed per individual experimental group. A total of 3 independent experiments with 4 doses of semen each was performed.

### Experiment 3: effect of porcine relaxin (pRLX) on sperm viability after storage

On Day 0, Day 1, Day 2, or Day 4, semen aliquots were prepared and purified spermatozoa were treated with 0 or 100 ng pRLX/ml, as mentioned above. After incubation of 1 h at 37°C, sperm motility was assessed and data were comparable to those obtained in experiment 2a. Therefore, aliquots of spermatozoa were collected and stained for either plasma membrane (Propidium Iodide; Sigma-Aldrich Co., Saint Louis, MO, USA; 5 min, 5 μg/ml) or mitochondria membrane (JC-1; Cayman Chemical Co., Ann Arbor, MI, USA; 20 min, 1 μM) integrity assessments. In both staining procedures, samples were incubated at 37°C and then centrifuged (250 g - 5 min) to eliminate the excess of dyes. The proportions of viable or membrane intact spermatozoa were evaluated with a flow cytometer (Becton Dickinson FACSCalibur™) set for 5,000 total events per analysis.

### Experiment 4: effect of relaxin (pRLX) on cAMP content of spermatozoa after storage

Spermatozoa were prepared and incubated with relaxin (0, 50, and 100 ng/ml) as specified in experiment 2a. Treated spermatozoa were collected at different time-points (Day 0, Day 1, and Day 2) and centrifuged (250 g - 5 min) and pellets were stored at −20°C until analysis. Cyclic AMP (cAMP) contents of spermatozoa were evaluated using the Direct Cyclic AMP Enzyme Immunoassay kit (Assay Designs Inc., Ann Arbor, MI). Briefly, frozen-thawed pellets of spermatozoa were treated with 0.1 M HCl (30 min) and lysed cells were centrifuged (600 g - 15 min). Supernatants were recovered and subsets of 100 μl, corresponding to approximately 7 × 10^6^ sperm cells, were immediately used for acetylated cAMP assays as recommended by the manufacturer. The assay is a colorimetric method that generates a yellow color read on a Spectramax Plus microplate spectrophotometer (Molecular devices, Sunnyvale, CA) at 405 nm. Obtained optical densities were used to calculate the cAMP concentrations of samples, using a standard curve. The detection limit of the kit was less than 5 femtomoles/sample. As a positive control, spermatozoa treated with forskolin (250 μg/ml; Sigma Aldrich, Saint Louis, MO), to induce cAMP production within cells, were assayed per the manufacturer’s protocol for acetylated samples (Cyclic AMP EIA kit; Cayman Chemical, Ann Arbor, WI). Sperm cells were collected on Day 0 and Day 2 for cAMP assays, following 15 and 60 minutes incubation with forskolin at 37°C. All procedures were done at room temperature and data were expressed as the averages (+/− SD or SEM) of three independent semen collections ran in triplicates for each treatment group (n = 9 reads/treatment group).

### Statistical analyses

Experiments were performed in three to four independent replicates, using three to four doses of pooled semen. Sperm motility characteristics per experimental condition were analyzed from 36 to 48 fields of micro-chamber-slides. Data were statistically analyzed using the GLM procedures of SAS®, version 9.2 (SAS Institute Inc., Cary, NC). The homogeneity of distribution (Kolgomorov Smirnov test) and variance (Levene’s test) were performed, followed by the two-way analyses of variance (relaxin or storage × experimental replicate) to evaluate the impacts of relaxin and each storage day on all dependent variables (motility and velocity parameters, cAMP content, plasma membrane and mitochondria membrane integrity). The effects of storage day were evaluated with ANOVA repeated measures. When necessary, a Fisher’s Least Square Difference (LSD) test was performed for pairwise comparisons. The threshold of statistical significance was fixed at *p* values less or equal to 0.05.

## Results

### Experiment 1: immunofluorescence detection of RXFP1 and RXFP2 receptors

**Figure 1:**
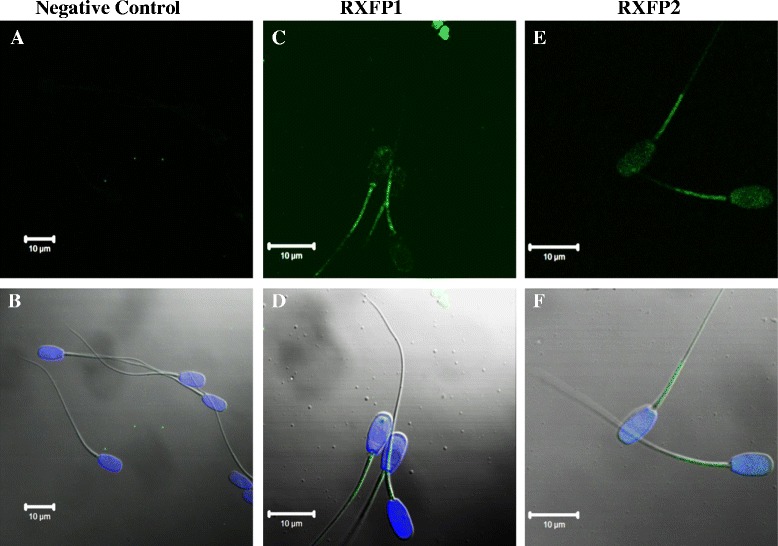
**Representative micrographs of immunofluorescence detection of RXFP1 and RXFP2 relaxin receptors in boar spermatozoa.** The immunofluorescence detection of RXFP1 and RXFP2 (green FITC-staining) receptors of boar spermatozoa are shown in micrographs **C** and **E**, respectively. The micrograph **A** corresponds to the negative control, without a distinctive green FITC staining on spermatozoa. The bottom panel shows overlay images of visible light, blue staining of sperm nuclei (DAPI), and green FITC-staining of the Control **(B)**, RXFP1 **(D)** and RXFP 2 **(F)** groups. Scale bars correspond to 10 μm.

Fluorescence signals indicated the presence of RXFP1 and RXFP2 proteins on boar spermatozoa (Figure 1). Both receptors were detected on the entire spermatozoon and higher accumulation was observed in the mid-piece region of all spermatozoa. However, the RXFP1 fluorescence signal was generally stronger than that of RXFP2, especially in the tail and often in the neck region of spermatozoa. The RXFP2 signal appeared more homogenous in the head.

### Experiment 2a: effect of porcine relaxin (pRLX) on sperm motility after short storage

The effect of pRLX (0, 50 and 100 ng/ml) on the global motility of spermatozoa during storage (Day 0, Day 1, and Day 2 post-collection) is summarized in Table 1. There were no significant effects of pRLX added on Day 0 (*p > 0.05*). As expected, these proportions gradually and significantly decreased during storage in the control group, and the beneficial effect of relaxin on the proportions of motile spermatozoa was observed at each storage time. At each storage time-point, the positive effect of relaxin was characterized with the slowed-pace of the decline and the maintenance of higher proportions of motile spermatozoa, in comparison to the control group (*p < 0.05*). The marked effect of pRLX was observed on Day 1 (*p < 0.05*).

**Table 1:** **Effect of porcine relaxin (pRLX) on sperm motility and velocity parameters (VAP, VSL, and VCL) following storage**

**Relaxin concentrations (ng/ml)**	**Day 0**	**Day 1**	**Day 2**
	Proportions of total motile spermatozoa (%)		
0	62.7 +/− 2.7^a, ¥^	58.5 +/− 2.5^a, §^	54.1 +/− 3.2^a, €^
50	65.3 +/− 2.9^a, ¥^	62.3 +/− 2.5^ab, ¥^	57.1 +/− 2.9^a, §^
100	65.4 +/− 2.3^a, ¥^	66.1 +/− 1.9^b, ¥^	56.8 +/− 3.1^a, §^
	Proportions of progressive spermatozoa (%)		
0	23.1 +/− 2.4^a, ¥^	30.8 +/− 2.3^a, §^	26.2 +/− 2.4^a, ¥§^
50	28.2 +/− 4.2^ab, ¥^	38.8 +/− 2.0^b, §^	32.2 +/− 2.1^b, ¥§^
100	32.3 +/− 1.9^b, ¥^	36.6 +/− 1.9^b, ¥^	33.4 +/− 2.5^b, ¥^
	Proportions of rapid spermatozoa (%)		
0	32.5 +/− 3.9^a, ¥^	46.2 +/− 2.6^a, §^	41.6 +/− 4.3^a, §^
50	35.1 +/− 4.8^ab, ¥^	48.4 +/− 3.0^a, §^	48.0 +/− 2.6^b, §^
100	40.4 +/− 2.4^b, ¥^	49.1 +/− 2.9^a, §^	48.9 +/− 2.7^b, §^
	Average Path Velocity (VAP; μm/s)		
0	61.4 +/− 3.1^a, ¥^	71.1 +/− 2.5^a, §^	72.7 +/− 3.3^a, §^
50	65.8 +/− 3.7^ab, ¥^	73.1 +/− 2.9^a, ¥§^	76.2 +/− 3.2^a, §^
100	69.8 +/− 2.9^b, ¥^	74.0 +/− 1.7^a, ¥§^	76.2 +/− 2.8^a, §^
	Curvilinear Velocity (VCL; μm/s)		
0	119.4 +/− 5.7^a, ¥^	142.3 +/− 4.4^a, §^	146.5 +/− 4.6^a, §^
50	120.3 +/− 4.7^a, ¥^	139.5 +/− 4.9^a, §^	145.6 +/− 4.7^a, §^
100	125.2 +/− 5.6^a, ¥^	137.5 +/− 2.9^a, §^	138.4 +/− 4.1^a, §^
	Straight Line Velocity (VSL; μm/s)		
0	48.0 +/− 1.9^a, ¥^	52.7 +/− 1.7^a, §^	53.7 +/− 2.5^a, §^
50	53.2 +/− 3.0^ab, ¥^	57.5 +/− 2.0^ab, §^	57.0 +/− 2.4^a, §^
100	57.1 +/− 2.1^b, ¥^	58.6 +/− 1.2^b, ¥^	56.9 +/− 2.3^a, ¥^

Letters (a,b,c) and symbols (¥,§,€) indicate significant differences within the same column (relaxin effect) and the same row (storage effect), respectively (*p* ≤ 0.05). Data are means (+/− SEM) of 4 independent replicates, representing approximately 8,869 to 10,600 spermatozoa analyzed on a total of 48 CASA fields.

The proportions of progressive and rapid spermatozoa in the control group steadily increased during storage (*p > 0.05*). The presence of pRLX significantly affected these proportions, regardless of the storage day (*p < 0.05*; Table 1). The beneficial effect of relaxin was constant on the progressive spermatozoa over the storage time, while its distinct effect was observed on Day 2, on rapid spermatozoa (*p < 0.05*).

**Figure 2:**
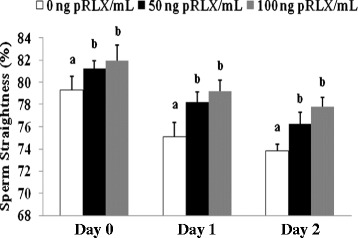
**Effect of pRLX on sperm straightness (VSL/VAP × 100).** Data are mean percentages (+/− SD) of 4 independent replicates, with 48 individual observations or micro-chamber fields during the CASA analysis. Columns with different letters (a, b) differ significantly within the same day (p ≤ 0.05). The presence of pRLX slowed the pace of the straightness decreased of spermatozoa during storage (p < 0.05).

**Figure 3:**
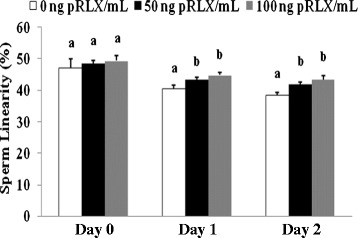
**Effect of pRLX on sperm linearity (VSL/VCL × 100).** Data are mean percentages (+/− SD) of 4 independent experiments, with 48 individual observations or micro-chamber fields during the CASA analysis. Columns with different letters (a, b) differ significantly within the same day (p ≤ 0.05). The presence of pRLX significantly decelerated the pace of sperm linearity decline during storage (p ≤ 0.05).

The effect of relaxin on sperm velocity parameters are summarized in Table 1, Figures 2 and 3. Regardless of the group, each of these parameters was significantly increased during storage (*p < 0.05*). Both VAP and VSL parameters significantly increased in the presence of 100 ng/ml pRLX on Day 0. This effect persisted until Day 1 with VSL only (*p < 0.05*), while 50 ng/ml had no significant effects at all (*p > 0.05*). The straightness (VSL/VAP) of spermatozoa in the control group significantly decreased during storage (*p < 0.05*; Figure 2), and the supplementation of sperm suspensions with pRLX maintained the straightness values at significantly higher levels during storage, when compared to the control group of corresponding storage days (*p ≤ 0.05*). However, the gradual loss of sperm straightness observed from Day 1 was not fully restored by the presence of pRLX (*p < 0.05*). Furthermore, the linearity (VSL/VCL) of spermatozoa progressively decreased in the control group during storage (*p < 0.05*; Figure 3) and the presence of pRLX partially attenuated the decline of the sperm linearity on Day 1 and Day 2 (*p ≤ 0.05*). The ALH and BCF values in the control groups varied respectively, from 4.8 μm (+/− 0.2) and 39 Hz (+/− 0.7) on Day 0 to 5.9 μm (+/− 0.1) and 41 Hz (+/− 0.5) on Day 2. The ALH values significantly increased in all groups during storage (*p < 0.05*), while the BCF data remained comparable regardless of storage days and relaxin concentrations (*p > 0.05*).

### Experiment 2b: effect of porcine relaxin (pRLX) on sperm motility after longer storage

Experiment 2a revealed a limited or no effect of pRLX on Day 2 of storage. Relaxin effects were evaluated at both 100 and 500 ng/ml on Day 2 and 4. The effects of 100 ng/ml pRLX were comparable to the control group at each storage day (*p > 0.05*), while 500 ng/ml had significant effects on many analyzed parameters (*p < 0.05*). Data obtained on both Day 2 and Day 4 were similar and only Day 4 data are shown in Table 2. The supplementation of sperm suspensions with 100 or 500 ng/ml pRLX after storage (Day 4) did not significantly affect the proportions of motile, linear (LIN), and straight (STR) spermatozoa. However, the proportions of progressive and rapid spermatozoa, as well as the VAP, VCL, and VSL parameters were significantly increased (*p < 0.05*; Table 2).

**Table 2:** **Effect of porcine relaxin (pRLX) on sperm motility and velocity parameters following a longer storage (Day 4 post-semen collection)**

**Parameters**	**Relaxin concentrations (ng/ml)**			**P**
	**0**	**100**	**500**	
Motility (%)*	66.8 +/− 2.6^a^	67 +/− 2.3^a^	68.3 +/− 1.4^a^	0.71
Progressive (%)**	51.4 +/− 1.9^a^	53.4 +/−3.4^a^	58.0 +/− 1.2^b^	0.03
Rapid (%)**	68.4 +/− 1.9^a^	69 +/− 2.7^a^	75.1 +/− 1.2^b^	0.02
VAP (μm/s)	80.5 +/− 2.3^a^	79.5 +/− 2.7^a^	90.2 +/− 1.5^b^	0.03
VSL (μm/s)	51.5 +/− 1.8^a^	55.2 +/− 3.4^a,b^	57.6 +/− 1.4^b^	0.04
VCL(μm/s)	175.0 +/− 3.6^a^	172 +/− 4.6^a^	183.7 +/− 2.3^b^	0.05
STR (VSL/VAP, %)	61.0 +/− 0.9^a^	62 +/− 2.6^a^	61.4 +/− 0.7^a^	0.99
LIN (VSL/VCL, %)	29.6 +/− 0.6^a^	32 +/− 1.7^a^	31.7 +/− 0.6^a^	0.39

Asterisks indicate proportions from total (*) or motile (**) spermatozoa. Letters (a,b) indicate significant differences within the same line (*p* < 0.05). Data are means (+/− SEM) of 4 independent replicates, representing approximately 7,200 +/− 256 spermatozoa analyzed on a total of 36 CASA fields.

### Experiment 3: effect of porcine relaxin (pRLX) on sperm viability after storage

**Figure 4:**
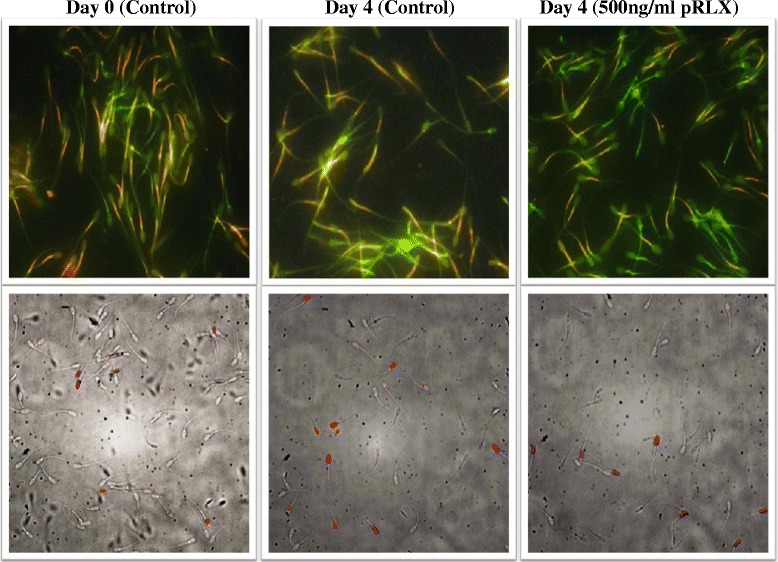
**Fluorescence microscopy evaluation of sperm viability after storage.** Representative images show spermatozoa stained for mitochondria membrane potential (JC-1; Upper panel) or plasma membrane integrity (PI; Lower panel) evaluation. Upon excitation at 488 nm, spermatozoa with intact (high potential) or damaged (low potential) mitochondrial membranes fluoresce red-orange or green, respectively, while those with damaged plasma membrane fluoresce red. Magnification 200X.

Obtained data are mean proportions (+/− SEM) of 4 independent replicates, with a total of 5,000 cells analyzed in each replicate. The proportions of spermatozoa with intact plasma (82 +/− 3 %) and mitochondrial (88 +/− 1.3 %) membranes remained unchanged during storage (*p > 0.05*; Table 3) and relaxin supplementation to sperm suspensions had no significant effects (86 +/− 3 % and 88 +/− 1.3 %, respectively; *p > 0.05*). The mean fluorescence intensity that is indicative of the extent of membrane damages significantly increased in PI-stained spermatozoa during storage (*p < 0.05*), and the presence of relaxin could not prevent this increase (*p > 0.05*). Neither the storage time nor the presence of relaxin affected the mean fluorescence intensity of JC-1-stained cells (*p > 0.05*). Representative fluorescent images of PI- and JC1-stained spermatozoa on Day 0 and Day 4 are shown in Figure 4.

**Table 3:** **Effect of porcine relaxin (pRLX) on sperm viability following storage**

**Relaxin concentrations (ng/ml)**	**Day 0**	**Day 1**	**Day 2**	**Day 4**
	Spermatozoa with intact plasma membrane (%)			
0	85.5 +/− 5.5^a, ¥^	85.5 +/− 5.5^a, ¥^	79.0 +/− 5.5^a, ¥^	79.5 +/− 5.5^a, ¥^
100	87.0 +/− 5.5^a, ¥^	90.5 +/− 5.5^a, ¥^	87.5 +/− 5.5^a, ¥^	80.5 +/− 5.5^a, ¥^
	Mean fluorescence of PI in all sperm cells (RFI)			
0	41.0 +/− 7.1^¥§^	26.0 +/− 7.1^¥^	56.5 +/− 7.1^§€^	86.0 +/− 7.1^a, €^
100	39.5 +/− 7.1^¥§^	26.0 +/− 7.1^¥^	52.5 +/− 7.1^§^	38.5 +/− 7.1^b, ¥§^
	Spermatozoa with intact mitochondrial membrane (%)			
0	88.5 +/− 2.5^a, ¥^	88.0 +/− 2.5^a, ¥^	89.0 +/− 2.5^a, ¥^	88.0 +/− 2.5^a, ¥^
100	89.0 +/− 2.5^a, ¥^	88.5 +/− 2.5^a, ¥^	84.0 +/− 2.5^a, ¥^	90.0 +/− 2.5^a, ¥^
	Mean fluorescence of JC-1 in all sperm cells (RFI)			
0	451 +/− 318^a, ¥^	1254 +/− 318^a, ¥^	682 +/− 318^a, ¥^	802 +/− 318^a, ¥^
100	583 +/− 318^a, ¥^	1338 +/− 318^a, ¥^	1290 +/− 318^a, ¥^	748 +/− 318^a, ¥^

Letters (a,b) and symbols (¥,§,€) indicate significant differences within the same column (relaxin effect) and the same row (storage effect), respectively (p < 0.05). Data are means (+/− SEM) 4 independent replicates, with 5,000 cells analyzed in each experimental condition per replicate. The global effects of relaxin on sperm membrane integrity, mean fluorescence of PI (RFI), mitochondrial membrane integrity, and mean fluorescence of JC-1 were 0.333, 0.03, 0.788, and 0.417, respectively. The global effect of storage on sperm membrane integrity, mean fluorescence of PI (RFI), mitochondrial membrane integrity, and mean fluorescence of JC-1 were 0.519, 0.004, 0.760, and 0.172, respectively.

### Experiment 4: effect of relaxin (pRLX) on cAMP content of spermatozoa after storage

**Figure 5:**
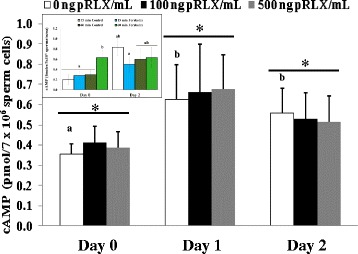
**Effect of pRLX on sperm cAMP content after storage.** Data are means (+/− SD) of three independent replicates, corresponding to nine individual analyses. The cAMP levels of spermatozoa in the control groups (0 ng pRLX/ml) significantly increased during storage (a,b; p < 0.05) and no significant effect of pRLX was found within the same storage day (*; p > 0.05). The insert indicates groups of spermatozoa ran independently to evaluate the effect of forskolin (250 μg/ml), used as positive control for cAMP assessment on Day 0 and Day 2. Data are mean+/− SEM and columns with different letters **(a, b)** indicating significant differences within the same Day.

Data summarized in Figure 5 are means +/− SD of nine observations per experimental condition. The lowest concentration of relaxin detected in spermatozoa (37 fmol per 7 × 10^6^ sperm) was well above the sensitivity limit of the detection kit (<0.5 fmoles per sample). In comparison to Day 0, the cAMP contents of spermatozoa in the control groups were significantly increased during storage with the highest levels found on Day 1 (*p ≤ 0.05*). However, there were no significant differences between groups on the same day of storage, with neither 100 nor 500 ng/ml pRLX having significant effect on the cAMP levels (*p > 0.05*). As expected, the presence of forskolin induced a significant increase of intraspermatic cAMP that was only observed on Day 0 after 60 minutes incubation (63 +/− 0.15 vs. 30 +/− 0.1 fmoles per 3 × 10^6^ sperm to the control; *p < 0.05,* See Insert in Figure 5).

## Discussion

The presence of relaxin in male reproductive organs is widely reported in various species and the association of the seminal plasma relaxin with sperm motility has been confirmed in numerous studies [11,13,16]. Here, we tested and confirmed the potential of relaxin to sustain, restore, and/or improve the motility characteristics of boar spermatozoa after prolonged storage in a commercial extender. We also showed for the first time with a direct approach, the presence of relaxin membrane receptors RXFP1 and RXFP2 through which relaxin may exert its effects on boar spermatozoa. However, we were not able to attest whether the stimulation of these receptors with relaxin elicits cAMP signaling cascade, as reported in a previous study using human sperm [25].

To date, studies that investigated relaxin receptors on boar spermatozoa utilized indirect approaches based upon labeled relaxin. In a previous study, we showed, through different technical approaches, namely immunofluorescence and western-immunoblotting, that antibodies raised against human RXFP1 and RXFP2 could be used to successfully detect both receptors in pig ovaries (follicles and corpora lutea) [26]. These antibodies targeted receptors’ amino acid sequences (or epitopes) that are highly similar across species such as *Bos taurus, Canis familiaris, Equus cabalus, Homo sapiens, Mus musculus,* and *Pan troglodyte*. Here we used the same antibodies for immunofluorescence detection of RXFP1 and RXFP2 receptors in boar spermatozoa. The current observations confirm our previous report using western-immunoblotting [26], as well as other studies published in boar [14] and human [31] spermatozoa using indirect approaches that consisted in the labeling of relaxin to target unspecified binding sites. To the best of our knowledge, the present report is the first to illustrate relaxin receptors on boar spermatozoa through a direct detection. The findings suggest that the used anti-human RXFP1 and RXFP2 antibodies could be effective in pigs despite the smaller sizes of protein targets observed in a previous study after westernimmunoblotting [26]. However, it should be kept in mind that these antibodies were chosen based upon the similarities between RXFP1 or RXFP2 immunogenic amino acid sequences of various species using bioinformatics tools. Therefore, additional studies using different sources of antibodies will be needed for further confirmation of the current observations.

In the present study, both relaxin receptors appeared differentially accumulated and distributed on the spermatozoon, which may likely be correlated to their participation in various biological processes following activation of receptors located on the tail and the neck (e.g., for sperm motility) or on the head (e.g., for interactions with immediate environment such as the cervix [17]). The RXFP1 distribution on pig spermatozoa showed slight differences with their human counterparts [25], which may indicate a possible species-specificity. As to the differential localization of both receptors on pig spermatozoa, it likely that this does not have dramatic impacts on the well-documented effects of exogenous relaxin on events such as, enhanced sperm motility, capacitation, acrosome reaction, and incidence of DNA fragmentation [23,25], which could be elicited by one or both receptor types. Hence, the development of powerful methods for direct detection of each receptor will be crucial for further elucidations of the involved intracellular mechanisms [25].

In light of the multitude biological effects of relaxin, which beneficial impact could be valuable in assisted reproductive technologies, we sought to dig into its potential effect on aged spermatozoa. Currently, boar semen is optimally conserved between 16°C and 18°C in appropriate extenders, but the motility and fertility potential of spermatozoa still decrease during prolonged storage. The maintenance of such temperatures in situations of delayed inseminations is not always guarantied and semen are often destroyed or transiently stored at room temperature until their use. This precarious storage condition contributes to the fast fertility decline of diluted semen, which led us to speculate that the supplementation of such semen doses with relaxin may help maintain or restore their quality and viability. In this study, relaxin concentrations were chosen to mimic those encountered by spermatozoa during their journey within the male and female genital tracts. Various reports in humans and pigs indicate that relaxin concentrations vary from 0.2 to 480 nM (≈1.1 to 3,000 ng/ml) in the seminal and utero-oviductal fluids [16,19,25,32,33], and concentrations of 1 to 10,000 ng relaxin/ml have been used in *in vitro* studies [14,34]. For examples, concentrations of 10 to 100 ng/ml improve the quality of murine [35] and porcine [34,36] oocytes, as well as motility of human [30] and pig [23,24] spermatozoa. Our study shows that relaxin concentrations, up to 500 ng/ml, enhance or maintain the motility of boar spermatozoa at levels that are comparable to unexposed control samples during storage (2 to 4 days post collection). These findings are in agreement with previous *in vitro* [23,24], and *in vivo* [11,19,21] studies, and most importantly with prior studies observing similar effects of relaxin (167 to 1,000 ng/ml) on motility of up to 5 hour-aged and washed human [22] and pig [21] spermatozoa. It is noteworthy to mention that the percoll selection and washing of living spermatozoa in this study were meant to ensure that the exogenous relaxin only (or mainly) interact with spermatozoa. Hence, our study brings additional evidences that highlight the positive effects of exogenous relaxin on prolonged and risky stored semen.

At present, compelling reports suggest that the beneficial effects of relaxin are concomitant with the semen quality. Studies conducted in human did not find any effects on high quality fresh semen [37,15] using relaxin concentration ranges of 10 to 100 nM (~62.5 to 600 ng/ml), while others found beneficial effects [25] that were mainly observed in aged and poor semen [37,15]. Therefore, the lack of relaxin effect on Day 0 (day of semen collection) remains unsurprising in our study, as semen were harvested from high fertile sires used for commercial purpose. Interestingly, we observed favorable effects of relaxin on other motility (progressive and rapid sperm) and velocity (VAP and VSL) parameters that were not previously reported on fresh spermatozoa (Day 0). These motility characteristics are important for the fertilizing potential of spermatozoa and our study shows that they constitute, together with the sperm linearity and straightness, the hallmarks of relaxin effects on stored/aged boar semen. The importance of these findings is supported by earlier reports in human indicating positive correlations between sperm straightness, straight line velocity (VSL) and fertilization rates [38,39]. In the present study, relaxin maintains both straightness and linearity at higher levels and limits the extent of the plasma membrane damage during storage, which may contribute to enhancing sperm effectiveness and smooth displacement within the utero-oviductal tube.

The binding of relaxin to its receptors is expected to activate cAMP for an overproduction of energy (ATP) [40,41] that is necessary for sperm motility. In our study, the lack of cAMP increase in the presence of relaxin was unexpected, but appeared in agreement with a prior study using human spermatozoa [22]. Nonetheless, these authors found that caffeine, rather than relaxin, stimulated human spermatozoa through the cAMP activation. Similar observations were seen in the current study with forskolin, but not relaxin, inducing a significant accumulation of cAMP after 1 hour incubation. Thus, we speculated that the absence of cAMP accumulation in the presence of relaxin was due to a likely difference in induced signaling cascades in boar spermatozoa or to a rapid and transient production that we may have missed. Indeed, a cAMP accumulation has been observed within 5 to 15 minutes, following exposure of human spermatozoa to relaxin [25]. These time-points were not chosen in our study because of the overall experimental design and thus, additional studies are commended to further characterize the relaxin cascade signal within the porcine spermatozoa.

## Conclusions

The study provides a direct evidence of the presence of RXFP1 and RXFP2 membrane receptors on boar spermatozoa, through which relaxin may exert its effects. Although the intra-spermatic signaling cascade triggered by relaxin remains to be elucidated in boar spermatozoa, the results suggest a potential use of relaxin as an effective supplement in aged or prolonged storage of semen for assisted reproduction. Indeed, the positive effects of seminal relaxin on freshly ejaculated spermatozoa may be lost under the modern assisted reproduction, due to high dilution of semen for artificial insemination dose preparations or to its inactivation/elimination following semen freezing-thawing procedures. Therefore, the addition of relaxin to fresh (or extended) [17] or frozen-thawed [42] semen prior artificial insemination may be of reproductive and commercial importances. Nonetheless, whether the positive effects of exogenous relaxin on motility parameters defining sperm progressiveness (straightness, linearity and VSL) during storage could translate into satisfactory field fertility remains to be elucidated.

## Additional files

Additional file 1: Figure S1. Multi-sequence alignment of the human RXFP1 immunogenic sequence. The antibody recognized an immunogenic sequence within the first 300 amino acids of the N-terminal region of the human RXFP1 protein. This sequence (Red colored) was aligned with reference sequence proteins of various species retrieved from the NCBI repository data base. Aligned sequences appeared highly conserved across species with great number of identical residues (*) and conserved (:) or semi-conserved (.) substitutions in the same column. Additional file 2: Figure S2. Multi-sequence alignment of the human RXFP2 immunogenic sequence. The antibody recognizes a sequence within the first 300 amino acids of the N-terminal region of the human RXFP1 protein. This sequence (red colored) was aligned with reference sequences of various species retrieved from the NCBI repository data base. Aligned sequences appeared highly conserved across species with great number of identical residues (*) and conserved (:) or semi-conserved (.) substitutions in the same column.

## Abbreviations

ANOVA: Analysis of variances
cAMP: Cyclic Adenosine MonoPhosphate
DNA: Deoxyribonucleic acid
FACS: Fluorescence-assisted cell sorting
FITC: Fluorescein isothiocyanate
IgG: Immunoglobulin gamma
JC-1: Tetraethylbenzimidazolylcarbocyanine iodide
PVP: Polyvinylpyrrolidone
RXFP: Relaxin family peptide receptors
