# Supplementary material for: Beneficial effects of relaxin on motility characteristics of stored boar spermatozoa
Source: Reprod Biol Endocrinol. 2015 Mar 31;13:24. doi: 10.1186/s12958-015-0021-4 (PMC4393568; doi:10.1186/s12958-015-0021-4)
Supplement: Additional file 1: Figure S1. — Multi-sequence alignment of the human RXFP1 immunogenic sequence. The antibody recognized an immunogenic sequence within the first 300 amino acids of the N-terminal region of the human RXFP1 protein. This sequence (Red colored) was aligned with reference sequence proteins of various species retrieved from the NCBI repository data base. Aligned sequences appeared highly conserved across species with great number of identical residues (*) and conserved (:) or semi-conserved (.) substitutions in the same column. [file 12958_2015_21_MOESM1_ESM.pdf]

```
                                                                               Length (AA)
Homo sapiens (NP_067647.2):       MTSGSVFFYILIFGKYFSHGGGQDVKCSLGYFPCGNITKCLPQLLHCNGVDDC-------   60
Mus musculus (NP_997617.1):       MTSGPFFFCIFIIGKYFTLGSAQDVSCPLGSFPCGNMSRCLPQLLHCNGVDDC-------   60
Bos taurus (XP_610789.3):         MTSRPVFLYILIFGKYFAPGRAQNAPCDLGYFPCGNTTKCLPQQLQCNGVDDC-------   60
Canis familiaris (XP_532702.2):   MSAAPVVIAARLGATKMPLG----------------------GYMQAKGLDSALKGSQLL   60
                                  *:: ...:   :..  :. *                        ::.:*:*..

Homo sapiens (NP_067647.2):       --------------------GNQADEDNCGDNNGWSLQFDKYFASYYKMTSQYP-FEAET  120
Mus musculus (NP_997617.1):       --------------------GNRADEDHCGDNNGWSLQLDKYFANYYKLASTNS-FEAET  120
Bos taurus (XP_610789.3):         --------------------ENHVDEDNCGDINGWSTQFDRYYGNNYKMTSLYPSIVAET  120
Canis familiaris (XP_532702.2):   SQRRPRGPLRARHWAEGPRQGRELRARGPGDNNGWPLQFDKYVANYYRMTSSYP-FEAQT  120
                                                       ..      ** ***. *:*:* .. *:::*  . : *:*

Homo sapiens (NP_067647.2):       PECLVGSVPVQCLCQGLELDCDETNLRAVPSVSSNVTAMSLQWNLIRKLPPDCFKNYHDL  180
Mus musculus (NP_997617.1):       SECLVGSVPMHCLCRDLELDCDEANLRAVPSVSSNVTVMSLQRNFIRTLPPNGFRKYHEL  180
Bos taurus (XP_610789.3):         SECLVGSPPMQCICQGLELECDEINLRAVPSVSSNVTFMSLRRNLIRKLPPNVFKRYHGL  180
Canis familiaris (XP_532702.2):   SECLVGSVPMQCVCRGLEVDCDETNLRAVPSVSSNVTLMSLRWNLIRKLPPDGFRKYHNL  180
                                  .****** *::*:*:.**::*** ************* ***: *:**.***: *:.** *

Homo sapiens (NP_067647.2):       QKLYLQNNKITSISIYAFRGLNSLTKLYLSHNRITFLKPGVFEDLHRLEWLIIEDNHLSR  240
Mus musculus (NP_997617.1):       QKLCLQNNRIHSVSVSAFRGLRSLTKLYLSHNRITFLKPGVFEDLHRLEWLIIEDNHLSR  240
Bos taurus (XP_610789.3):         QTLCLQNNKIRSVSVYAFRGLYSLTKLYLSHNRITLLKPGVFEDLHRLEWLIIEDNHLNR  240
Canis familiaris (XP_532702.2):   QKLCLQNNKIRSIPIYAFRGLHSLTKLYLSHNRITFLKPGVFEDLHRLEWLIIEDNHLSR  240
                                  *.* ****:* *:.: ***** *************:**********************.*

Homo sapiens (NP_067647.2):       ISPPTFYGLNSLILLVLMNNVLTRLPDKPLCQHMPRLHWLDLEGNHIHNLRNLTFISCSN  300
Mus musculus (NP_997617.1):       ISPLTFYGLNSLILLVLMNNALTRLPDKPLCQHMPRLHWLDFEGNRIHNLRNLTFISCNN  300
Bos taurus (XP_610789.3):         ISPLTFYGLNSLILLVLMNNVLTRLPDKSLCQHMPRLHWLDFEGNHIHNLRNLTFISCSN  300
Canis familiaris (XP_532702.2):   ISPLTFYGLNSLILLALMNNVLTHLPDKPLCQYMPRLHWLDLEDNHIHNLRNLTFISCGN  300
                                  *** ***********.****.**:****.***:********:*.*:************.*
```
