# Supplementary material for: Beneficial effects of relaxin on motility characteristics of stored boar spermatozoa
Source: Reprod Biol Endocrinol. 2015 Mar 31;13:24. doi: 10.1186/s12958-015-0021-4 (PMC4393568; doi:10.1186/s12958-015-0021-4)
Supplement: Additional file 2: Figure S2. — Multi-sequence alignment of the human RXFP2 immunogenic sequence. The antibody recognizes a sequence within the first 300 amino acids of the N-terminal region of the human RXFP1 protein. This sequence (red colored) was aligned with reference sequences of various species retrieved from the NCBI repository data base. Aligned sequences appeared highly conserved across species with great number of identical residues (*) and conserved (:) or semi-conserved (.) substitutions in the same column. [file 12958_2015_21_MOESM2_ESM.pdf]

		Length (AA)
Homo sapiens (NP_570718.1):	MIVFLVFKHLFSLRLITMFFLLHFIVLINVKDFALTQGSMITPSCQKGYFPCGNLTKCLP	60
Pan troglodyte (XP_522654.2):	MIVFLVFKHLFSLRLITMFFLLHFIVLINVKDFALTQGSMITPSCQKGYFPCGNLTKCLP	60
Canis familiaris (XP_855603.1)	-----------------MFPLLHFIVLIDVKDFPLTEGSTITPLCQKGYFPCGNLTKCLP	60
Equus cabalus (XP_001493815.1):	----------------MAMFLLLHFAFLIDVKDYVLTDGSTITPLCQKGYFPCGNLTKCLP	60
Mus musculus (NP_536716.1):	-----------------MWLLLHVILLTEVKDFALADSSMVAPLCPKGYFPCGNLTKCLP	60
	*: ***. .* :***: *::.* ::* * **************	
Homo sapiens (NP_570718.1):	RAFHCDGKDDCGNGADEENCGDTSGWATIFGTVHGNANSVALTQECFLKQYPQCCDCKET	120
Pan troglodyte (XP_522654.2):	RAFHCDGKDDCGNGADEENCGDTSGWATIFDTVHGNANSVALTQECFLKQYPQYCDCKET	120
Canis familiaris (XP_855603.1)	RAFHCDGVDDCGNGADEDNCGDTSGWATIFGTVHGNANNVALTQECFLNQYPQPCDCKGT	120
Equus cabalus (XP_001493815.1):	RAFHCDGMNDCGNGADEENCGDTSGWATIFGTVHGNANNVALTQECFLNQYPQRCDCKET	120
Mus musculus (NP_536716.1):	RAFHCDGVDDCGNGADEDNCGDTSGWTTIFGTVHGNVNKVTLTQECFLSQYPQHCYCREN	120
	******* :********:********:***.*****.*.*:*******.**** * *: .	
Homo sapiens (NP_570718.1):	ELECVNGDLKSVPMISNNVTLLSLKKNKIHSLPDKVFIKYTKLKKIFLQHNCIRHISRKA	180
Pan troglodyte (XP_522654.2):	ELECVNGDLKSVPMISNNVTLLSLKKNKIHSLPDKVFIKYTKLKKIFLQHNCIRHISRKA	180
Canis familiaris (XP_855603.1)	ELECINADLRAVPVISSNTTLLSLKKNKIHSLPDKVFTKYTQLKQIFLQHNCITHISRKA	180
Equus cabalus (XP_001493815.1):	ELECVNAGLKSVPTISSNVTLLSLKKNKIRSLPNKVFVKYTELKKMYLQHNCIRHISRKA	180
Mus musculus (NP_536716.1):	ELECVKADLKAVPKVSSNVTLLSLKKNKIHRLPVKVFSRYTELRKIYLQHNCITHISRRA	180
	****::..*::** :*.*.**********: ** *** :**:*::::****** ****:*	
Homo sapiens (NP_570718.1):	FFGLCNLQILYLNHNCITTLRPGIFKDLHQLTWLILDDNPITRISQRLFTGLNSLFFLSM	240
Pan troglodyte (XP_522654.2):	FFGLYNLQILYLNHNCITTLRPGIFKDLHQLTWLILDDNPITRISQRLFTGLNSLFFLSM	240
Canis familiaris (XP_855603.1)	FFGLHNLQILYLSHNCITTLRPGVFKDLHQLTWLILDDNPITRISQQLFTGLKSLFFLSM	240
Equus cabalus (XP_001493815.1):	FFGLHNLQILYLNHNCITTLRPGVFKDLHQLTWLILDDNPITSISQRLFTGLNSLFFLSM	240
Mus musculus (NP_536716.1):	FLGLHNLQILYLSHNCITSLRPGIFKDLHQLAWLILDDNPITRISQKSFMGLNSLFFLPM	240
	*:** *******.*****:****:*******:********** ***: * **:*****.*	
